# Berotralstat in hereditary angioedema due to C1 inhibitor deficiency: first real-world evidence from a Canadian center

**DOI:** 10.3389/fimmu.2024.1339421

**Published:** 2024-01-22

**Authors:** Cindy Srinivasan, Bruce Ritchie, Adil Adatia

**Affiliations:** ^1^Faculty of Medicine, University of Alberta, Edmonton, AB, Canada; ^2^Division of Hematology, Department of Medicine, University of Alberta, Edmonton, AB, Canada; ^3^Division of Pulmonary Medicine, Department of Medicine, University of Alberta, Edmonton, AB, Canada

**Keywords:** hereditary angioedema, berotralstat, kallikrein, C1 inhibitor, immunodeficiency

## Abstract

**Background:**

Hereditary angioedema due to C1 inhibitor deficiency is a rare genetic condition that causes recurrent swelling with consequent functional impairment and decreased quality of life. Long-term prophylaxis (LTP) to prevent angioedema episodes is a key component of disease management. Berotralstat, an oral, once-daily plasma kallikrein inhibitor, was approved for LTP by Health Canada in 2022.

**Methods:**

We conducted a retrospective, real-world study investigating the effectiveness and adverse effects of berotralstat. Data on angioedema frequency, disease control, and adverse events were tabulated. Patient satisfaction with treatment was scored on a 5-point Likert scale, with 1 representing very unsatisfied and 5 representing very satisfied with therapy.

**Results:**

From June, 2022 and May, 2023, 8 patients with HAE type 1 or type 2 received berotralstat. Effectiveness data were available for 7 patients who continued the drug for at least 3 months, 4 of whom switched to berotralstat from plasma-derived C1 inhibitor LTP. In these 7 patients, the average number of attacks per month decreased from 3.3 to 1.6 (p<0.05), representing a ~52% reduction in attack frequency. Median angioedema control test score numerically improved from 8 to 13 (p=0.0781). Of the 8 patients who received berotralstat, 3 reported no adverse effects and 5 experienced gastrointestinal side effects, which were mild and transient in 3 and led to discontinuation in 1. Average treatment satisfaction was between satisfied and very satisfied at 4.3.

**Conclusion:**

Berotralstat is an effective agent for long-term prophylaxis in HAE. Most patients experienced no adverse effects or mild, transient gastrointestinal symptoms.

## Introduction

Hereditary angioedema (HAE) is a rare genetic disease that causes episodic cutaneous and submucosal swelling predominantly affecting the limbs, face, gastrointestinal tract, and upper airway (1). The most common form of HAE is due to decreased or dysfunctional serum C1 inhibitor (HAE-C1-INH type 1 and type 2, respectively), which is the main inhibitor of plasma kallikrein (PK) and activated coagulation factor XII in the contact activation pathway. PK cleaves the vasoactive peptide bradykinin from high-molecular weight kininogen (HMWK), and thus the loss of its negative regulator causes an overabundance of bradykinin and subsequent swelling in affected patients (2).

Long-term prophylaxis (LTP) to prevent angioedema episodes is the cornerstone of current HAE management. With the advent of modern, highly effective LTP therapy, the aim of treatment has become complete disease control and the normalization of patient lives (3). The 2019 International/Canadian HAE guideline recommends intravenous or subcutaneous plasma-derived C1 inhibitor (pd-C1) or lanadelumab, a monoclonal antibody targeting PK, as the first line LTP agents (4).

Berotralstat is a synthetic small molecule developed using structure-guided design to inhibit PK (5). It is an orally bioavailable drug that binds to the active site of the PK serine protease domain, thereby preventing HMWK cleavage. In 2021, the phase 3 APEX-2 study showed that berotralstat reduced the mean frequency of angioedema episodes by 44%, with half of the patients receiving the 150 mg dose achieving ∼70% reduction in attack frequency (6). The most common treatment-emergent adverse events were gastrointestinal (GI) side effects such as abdominal pain, reflux, and diarrhea. Berotralstat received Canadian regulatory approval in 2022. Herein, we describe the first real-world study of berotralstat use in Canada.

## Methods

We conducted a retrospective study of patients with HAE-C1-INH seen at our Angioedema Center of Reference and Excellence (ACARE) (7) who received berotralstat for LTP. Ethics approval was obtained from the University of Alberta Research Ethics Board. Consent for the study was waived given the retrospective design and infeasibility (some patients had relocated to different jurisdictions. Included patients were (∼18 years of age and filled at least one prescription for berotralstat 150 mg daily (the only dose available in Canada). All patients were diagnosed with HAE-C1-INH using a guideline-recommended diagnostic approach (3). Demographic data, previous LTP usage, attack frequencies in the previous 3 months, side effect reports, and angioedema control test (AECT) scores (8) were collected from clinic charts. The AECT is a well-validated patient‐reported outcome tool used to measure disease control in patients with recurrent angioedema (8, 9). AECT scores range from 0 to 16 (∼10 signifies well-controlled disease) (9), and the clinically important difference (MCID) for improvement is 3 (10).

Starting in 2023, all patients at our center were additionally asked to grade their overall satisfaction with their LTP on a five-point Likert scale (1 representing very unsatisfied and 5 representing very satisfied with treatment) as part of routine care, which was also collected when available. Patients switching from a different LTP agent were instructed to overlap the two treatments by 1 month. Given previous reports of transient GI effects from berotralstat (6), all patients were counseled on the possibility of treatment-emergent diarrhea, abdominal pain, and pyrosis and were instructed to take the drug with food and use over-the-counter treatments (e.g., loperamide or antacids) as a temporizing measure if required; details of such medication use were collected from charts as well.

Data were collected at baseline and six months post-drug initiation or at the last available follow-up if the patient discontinued the drug. Data analysis was performed using GraphPad Prism (v9). Demographic data were summarized using descriptive statistics. The change in the mean number of attacks per month pre- and post-berotralstat was analyzed using the paired t-test. The change in median AECT scores was analyzed using the paired Wilcoxon rank sum test. A significance level of 0.05 was used for all analyses.

## Results

Between June, 2022 and May, 2023, 8 patients with HAE-C1-INH were prescribed berotralstat. The average age was 39.0 years (standard deviation: 15.8), 7/8 were female, 6/8 had HAE-C1-INH type 1, and 4/8 switched from a different LTP therapy (**Table 1**). Patient P3 stopped the medication within 48 hours due to severe diarrhea and mild abdominal pain, which promptly resolved after treatment discontinuation. Follow-up data were available for the remaining seven patients.

**Table 1:** Baseline characteristics of patients treated with berotralstat and duration of treatment overlap in those previously treated with LTP.

Subject	Age	Sex	HAE type	Previous LTP	LTP Overlap
P1	47	F	1	IV pd-C1 inhibitor	25 days
P2	27	M	1	None	––
P3	23	F	1	None	––
P4	31	F	1	None	––
P5	51	F	1	None	––
P6	38	F	2	SC pd-C1 inhibitor.	0 days
P7	26	F	2	SC pd-C1 inhibitor	0 days
P8	69	F	2	IV pd-C1 inhibitor	0 days

**Figure 1:**
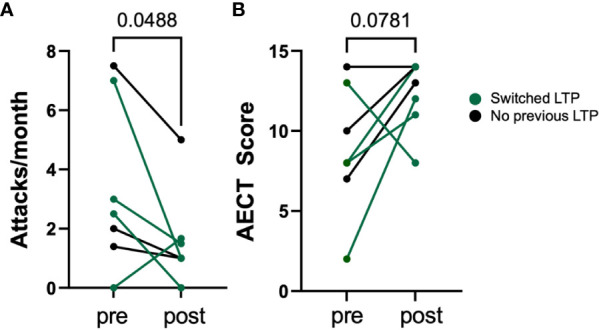
Change in angioedema attacks per month **(A)** and change in AECT score **(B)** in 7 HAE patients before and after using berotralstat. Mean number of attacks decreased significantly from 3.3 to 1.6 attacks per month (p<0.05, paired t-test).

The pre- and post-treatment angioedema attacks/month and AECT scores are shown in **Figure 1**. The mean number of attacks decreased significantly from 3.3 to 1.6 per month (p=0.0488). The median AECT score numerically improved from 8 to 13 (p=0.0781). In the four patients who switched from intravenous or subcutaneous pd-C1 LTP, the mean attack frequency decreased by 2.1 attacks/month, and the median AECT improved by 3.5 points. Patient P8 who switched from IV pd-C1 inhibitor LTP, discontinued berotralstat after 2 months of treatment due to an increase in attack frequency.

Of the 8 patients who received berotralstat, 3 reported no side effects. The remaining 5 reported GI side effects, which were mild (requiring no treatment) and lasted less than 1 week in 3/5. One patient (discussed above) discontinued the drug due to diarrhea, and one patient used proton pump inhibitor (PPI) treatment to manage pyrosis for one month and subsequently discontinued the PPI as the pyrosis resolved.

The average treatment satisfaction score for the 7 patients who continued treatment was between satisfied and very satisfied (4.3/5). Treatment satisfaction scores prior to starting berotralstat were not available.

## Discussion

In this retrospective, real-world study in patients with HAE-C1-INH, berotralstat reduced the mean attack frequency by 51.5% and numerically improved the median AECT by 5 points over 6 months. The observed effectiveness in patients who were switched from pd-C1 (intravenous or subcutaneous) was comparable to those who were LTP naïve. Satisfaction with treatment was high, and the drug was well tolerated with 75% of patients experiencing no adverse effects or mild, transient GI symptoms.

Our findings are similar to the results of real-world studies from other jurisdictions. A study from the United Kingdom (UK) reported effectiveness and adverse effects in 54 patients using patient surveys administered at one time point (with a recall period of up to 9 months) (11). They found a 64.9% reduction in attack frequency 4-6 months after starting berotralstat. A retrospective study in seven patients from Germany found that berotralstat reduced the mean attack frequency by 46.6% and improved the mean AECT by 3.56 points (12). Together, these data support the incorporation of berotralstat as a first line treatment option in clinical practice guidelines (12).

An important finding of our study is that despite counseling on the use of over-the-counter medications such as loperamide and antacids to manage GI side effects, only one of five patients who experienced such symptoms chose to do so. In three others, the symptoms were sufficiently mild and temporary that additional treatment was not sought, and in one patient, treatment was discontinued without a trial of such medicines. We observed a rate of discontinuation due to side effects of 1/8 (12.5%), which was comparable to the rate in the UK (9/54, 16.7%) and German (1/7, 14.3%) studies and perhaps somewhat higher than the discontinuation rate seen in the phase 3 study (7.5% for the 150 mg dose) (13).

The optimal protocol for transitioning patients to different LTP drugs has not been defined. The open-label APeX-S study included 13 patients who switched from pd-C1 (12 from SC and 1 from IV) to berotralstat (14), and co-treatment periods ranged from 0 to 4 months. However, the elimination half-life for IV and SC pd-C1 prophylaxis is short at 30.9 and 69 hours, respectively (15, 16), indicating that treatment overlap may be warranted. In our cohort, one of the three patients who did not overlap with pd-C1 experienced an increase in attack frequency leading to the discontinuation of berotralstat after 8 weeks of therapy.

A steady-state plasma concentration of berotralstat is achieved after 6-12 days, and the phase 3 study showed a reduction in mean attack frequency at 4 weeks (13). Hence, in the absence of more robust data, a co-treatment period of at least 2-4 weeks when switching from pd-C1 seems reasonable. Successful transition may also be aided by using the Berlin Protocol, which involves graded introduction of berotralstat to minimize side effects (17). An important unmet need remains identifying patients most likely to benefit from a transition to a kallikrein inhibitor. Genetic biomarkers, which can be used to predict disease severity (2, 18), may prove useful in this regard.

This study has limitations. Given the retrospective design, selection bias could have influenced the effectiveness and treatment satisfaction estimates. Treatment satisfaction scores were not available prior to the start of berotralstat, so pre- and post-drug comparisons were not possible. The size of the cohort analyzed was small, in keeping with the rarity of the disease. The short duration of follow-up may have underestimated the reduction in angioedema attack frequency as a further reduction was seen between 6 and 24 months of therapy in the phase 3 extension study (19). We suspect this is also the reason why the AECT score improvement did not achieve statistical significance. Future prospective, multicenter real-world studies would be helpful.

## Conclusion

Berotralstat is an effective and well-tolerated LTP agent. We suggest that berotralstat be incorporated into local clinical practice recommendations as a first-line treatment option alongside lanadelumab and pd-C1 and emphasize the consideration of patient values and preferences in choosing the LTP option that most closely aligns with their concept of normalization. Prospective studies are needed to determine the optimal transition protocol from one LTP drug to another.

## Data availability statement

The original contributions presented in the study are included in the article/supplementary material. Further inquiries can be directed to the corresponding author.

## Ethics statement

The studies involving humans were approved by University of Alberta Biomedical Ethics Board. The studies were conducted in accordance with the local legislation and institutional requirements. The ethics committee/institutional review board waived the requirement of written informed consent for participation from the participants or the participants’ legal guardians/next of kin because given the retrospective design and inability contact all patients.

## Author contributions

CS: Writing – original draft, Data curation, Visualization. BR: Conceptualization, Formal Analysis, Supervision, Writing – review & editing. AA: Writing – original draft, Conceptualization, Formal analysis, Methodology, Project administration, Resources, Supervision, Writing – review & editing.
